# Supplementary material for: Recent Advances in the Detection of Antibiotic and Multi-Drug Resistant Salmonella: An Update
Source: Int J Mol Sci. 2021 Mar 28;22(7):3499. doi: 10.3390/ijms22073499 (PMC8037659; doi:10.3390/ijms22073499)
Supplement: Supplementary file 1 [file ijms-22-03499-s001.pdf]

Table S1. Antibiotic resistant genes found by the CDC in eighteen Salmonella species sourced from food products (poultry, swine, beef) and humans.

Antibiotic	Resistant Genes	Salmonella spp
Quinolone	PMQR genes (oqxAB, aac (6')-Ib-cr), marRAB, soxRS, qnr, qepA	S. Typhimurium
Fluoroquinolone	gyr A, gyr B, A, topoisomerase IV	S. Typhimurium DT104
Gentamicin	aac, aadB, aac	S. Typhimurium
Streptomycin	strA/strB, aph(3)-Ib, aph (6)-Id	S. Typhimurium, S. Typhimurium DT204, S. Muenchen, S. Newport
Amoxicillin-Clavulanic Acid	blaCMY	S. Kentucky, S. Typhimurium DT104, S. Newport
Ceftiofur	blaCMY, blaCTX-M, blaCMY	S. Enteriditis, S. Infantis, S. Typhimurium, S. Heidelberg.
Ceftriaxone	blaCMY	S. Enteriditis, S. Infantis, S. Typhimurium, S. Heidelberg.
Sulfamethoxazole/Sulfisoxazole	sul1, sul2, sul 3	S. Bredeney, S. Kentucky, S. Enteriditis, S. Typhimurium, S. Muenchen, S. Newport, S. Hadar
Azithromycin*	AcrB*	S. Paratyphi A
Ampicillin	blaTEM, blaCMY, blaHERA	S. Kentucky, S. Typhimurium DT104, S. Muenchen, S.Newport,
Chloramphenicol	cat1,cat2, gyrA, qnrS, floR	S. Typhimurium DT104, S. Muenchen, S. Newport
Ciprofloxacin	qnrS	S. Poona
Nalidixic acid	qnrS	S. Poona
Tetracycline& Oxytetracycline	tetA, tetB, tetC, tetD, tetG, tetH, tetO, tetS, tetX, tetW, tet32,	S. Bredeney, S. Kentucky, S. Enteriditis, S. Typhimurium, S. Newport. S. Typhimurium DT104, S. Muenchen, S. Dublin, S. Agona, S. Choleraesuis, S. Heidelberg
Trimethoprim-sulfamethoxazole	dfrA	S. Typhimurium, S. Typhimurium DT104,S. Newport
Cefotaxime	blaTEM, blaSHV-1	S. Typhimurium
